# Correlates of screen time and mediators of differences by parental education among adolescents

**DOI:** 10.1186/s12887-020-02181-y

**Published:** 2020-06-05

**Authors:** Mekdes K. Gebremariam, Sigrun Henjum, Laura Terragni, Liv Elin Torheim

**Affiliations:** 1 Department of Nutrition, Institute of Basic Medical Sciences, University of Oslo, P.O.Box 1046, Blindern, 0317 Oslo, Norway; 2 Department of Nursing and Health Promotion, Faculty of Health Sciences, OsloMet – Oslo Metropolitan University, 0130 Oslo, Norway

**Keywords:** Sedentary behavior, Correlates, Mediators, Social inequalities, Adolescents

## Abstract

**Background:**

Existing literature shows that there is an inverse association between socioeconomic position and screen time among adolescents. What is less known is the mechanism behind these differences. The study aimed to explore individual, interpersonal and neighborhood environmental correlates of total screen time (TST) among adolescents and to assess their mediating role in the association between parental education and TST.

**Methods:**

A cross-sectional study including 706 adolescents (mean age of 13.6 (SD = 0.3)) was used to collect data at schools through an online questionnaire. Multiple regression analyses were used to explore factors associated with TST. Mediation analyses were conducted to assess whether these factors mediated the association between parental education and TST.

**Results:**

Multiple linear regression analyses, adjusted for gender and age, showed that parental modelling of TV and movie streaming, TV/movie streaming during dinner and access to screens were positively related to TST. Self-efficacy towards limiting TV and movie streaming, self-efficacy towards limiting computer/electronic game use, and the perceived opportunities for physical activity in the neighborhood were inversely related to total screen time. All of these factors except self-efficacy towards limiting TV and movie streaming mediated the association between parental education and TST.

**Conclusions:**

The study identified several modifiable factors at the individual, interpersonal and neighborhood environmental levels that can be targeted in interventions aimed at decreasing screen time among youth in general and among those with a low socioeconomic position in particular.

## Background

Research focusing on sedentary behaviors (SBs) has received increased attention in recent years due to concerns about excessive time spent sedentary in modern societies. Sedentary behavior is defined as any waking behavior characterized by an energy expenditure ≤1.5 METs while in a sitting or reclining posture [1]. Screen-based SBs are among these behaviors and are highly prevalent among youth [2]. SBs are associated with several adverse health impacts among youth [3, 4]. A review of 235 studies representing 1,657,064 unique participants from 71 different countries found that screen time and television (TV) viewing were positively associated with unfavorable body composition and higher clustered cardiometabolic risk scores. TV viewing and video game use were positively associated with unfavorable behavioral conduct. Screen time was inversely related to fitness and self-esteem [3]. Associations between SB and health outcomes are however not always consistent [4]. In addition, SBs track moderately from childhood and adolescence [5]; early interventions targeting these behaviors are thus vital. In order to inform such interventions, there is a need to identify key correlates of SBs. Existing evidence has identified parental modeling [6–8], parental rules [7, 9], the presence of a bedroom TV [6], more access to electronic devices [7, 9] and ethnicity [6, 7, 9, 10] as correlates of screen-based SBs among youth. A recent systematic review focusing on neighborhood environmental correlates of SB found that traffic, availability of a favorable environment for PA and higher residential density were associated with lower levels of SB among adolescents [11]; the authors however concluded that there were few studies investigating the association between SB and neighborhood characteristics, making the evidence limited [11]. There are indeed multiple levels of influence on health behaviors as postulated in the social ecological model of health behaviors including social, psychological and environmental influences [12]. The best approach to change behavior is through multi-level interventions, thus knowledge about important factors at these different levels of influence is vital [12]. The need to include more screen-based SBs including more contemporary screen activities has also been highlighted repeatedly in the literature.

In addition, socioeconomic differences in screen-based SB have been documented in several studies including Norwegian studies [6, 7, 9, 10, 13]. Such differences can lead to inequalities in SB-related adverse health outcomes. In this regard, a recent systematic review documented that screen-based SBs are consistent mediators of socioeconomic differences in body weight among youth [14]. Identifying mediators of socioeconomic differences in screen time would thus provide useful information for interventions aimed at tackling inequalities in obesity and other adverse health effects of SBs. A mediator represents an intervening variable in the causal pathway between exposure and outcome [15]. Socioeconomic inequalities related to factors such as education, income, employment and occupation might be associated with unequal exposure to risk factors and unequal access to health-promoting resources [16]. Thus, correlates of SBs at the different levels of the social ecological model that vary by socioeconomic position (SEP) have the potential to mediate socioeconomic differences in SB. The few existing studies exploring mediators of socioeconomic differences in SB included in a review identified the following mediators: frequency of eating dinner and snacks in front of TV, parental TV co-viewing and regulation, availability of media in bedroom and parental modelling [13]. Only one of these five studies used total screen time (TST) as an outcome measure [17]. More studies exploring mediators of socioeconomic differences in SB were called for, namely studies including a broader range of SBs and exploring the association between SEP and environmental correlates [13].

Against this background, the aim of the present study was to assess individual, interpersonal and perceived environmental correlates of TST among adolescents and to explore whether these correlates mediated parental educational differences in TST.

## Methods

### Design and sample

The participants in this study were pupils from eleven secondary schools participating in the Environmental determinantS of dietary BehaviorS among adolescENtS (ESSENS) cross-sectional study. All twelve secondary schools in the Øvre Romerike region located in the Eastern part of Norway were invited to participate in the study, and eleven accepted the invitation. In total, 1163 adolescents in the eighth grade were invited to participate in this study and a total of 781 (67%) received parental consent for participation. A total of 742 adolescents (64% of those invited and 95% of those with parental consent) participated in the study which was conducted at schools between October and December 2016.

### Data collection and measures

A web-based questionnaire was used to collect data from the adolescents. The questionnaires were filled in at school, and took approximately 30–45 min to complete. The questionnaire was pre-tested for clarity and length among a group of adolescents of the same age as the study participants (*n* = 23), prior to the main study.

#### Outcome variable

##### Screen-based sedentary behaviors

The following questions with pre-coded answer categories were used to assess screen time: How many hours do you usually watch TV, as well as DVDs, videos or movies on the PC, telephone or tablet in your spare time on a normal weekday? How many hours do you usually play computer games, games on a game console (e.g. PlayStation, Xbox, GameCube), games on a tablet or mobile phone on a normal weekday? How many hours do you usually use a computer, tablet or phone for activities such as chatting, internet, emailing, Facebook and Instagram on a normal weekday? The same questions were asked for a normal weekend day. The answer categories ranged from none to 4 h or more per day for all questions. Separate weekly scores for the different screen-based SB were calculated by summing hours reported for an average weekday (multiplied by five) and an average weekend day (multiplied by 2), and then summed to create a TST variable.

The screen time measures were adopted and modified (to reflect recent patterns in screen-based activities) from previous measures with evidence of moderate construct validity [18] and moderate test-retest reliability [18, 19].

#### Potential mediators

Self-efficacy towards watching TV, as well as DVD, videos or movies on phone or tablet was assessed using a five items scale (e.g. How sure are you that you can … limit watching TV as well as DVD, videos or movies on phone or tablet to 1 h at least one school day?). Self-efficacy towards use of computer/electronic games was assessed using a five items scale (e.g. How sure are you that you can … limit playing computer games including game consoles, games on mobile or tablet to 1 h at least one school day?). Both weekday and weekend screen use were included in the self-efficacy questions [20].

Screen viewing during meals was assessed using three questions about how often the adolescents watched TV, DVDs, video or movies on phone or tablet during breakfast, lunch and dinner. There were 5 response options ranging from “never” to “always”.

Parental modeling was assessed using the question: How often do your parents watch TV as well as DVDs, video or movies on phone or tablet? There were 5 response options ranging from “never” to “always”.

Parental co-viewing was assessed using the question: How often do you watch TV as well as DVDs, video or movies on phone or tablet together with your parents? There were 7 response options ranging from “never” to “every day, more than once/day”.

The questions assessing screen viewing during meals, parental modeling and parental co-viewing were adapted from previous measures with evidence of adequate test-retest reliability [18].

Access to screens was assessed using three questions: Do you have a TV in your bedroom? Do you have your own computer? Do you have your own tablet? The questions had a yes (1) and no (0) response options. The answers to the questions were summed up to create a variable assessing total access to screens.

Neighborhood safety was assessed using the question: It is safe to walk or play alone in my neighborhood during the day (there were 5 response options ranging from “totally agree” to “totally disagree”).

Perceived opportunities for PA in neighborhood was assessed using a Likert-type scale (There are places in the vicinity of my home I can go out and play; there are other children nearby home to go out and play with).

Neighborhood facilities for PA were explored using the statement: “there are playgrounds and parks near my home where I can play/be physically active” (5 response options ranging from “totally agree” to “totally disagree”).

The items assessing neighborhood safety, perceived opportunities for PA in neighborhood, neighborhood facilities for PA were adopted from measures developed by Ommundsen et al. [21].

#### Parental education

Information on parental education was gathered as part of the parental informed consent for the adolescent. It was categorized into: low (12 years of education or less, which corresponded to secondary education or lower) and high (13 years of education and more, which corresponded to university or college attendance). Educational status of the parent with the longest education or else the one available was used in the analyses.

### Statistical analyses

The analytical sample for this study was made up of 706 participants with information on parental education (36 participants with missing data on parental education were excluded). Since schools were the unit of measurement in this study, we checked for clustering effect through the Linear Mixed Model procedure. Only 1% of the unexplained variance in TST was at the school level. Hence, adjustment for clustering effect was not done. Descriptive analyses were first conducted. Univariable linear regression analyses were then used to explore factors at the individual, interpersonal, family and neighbourhood levels associated with TST. Thereafter, factors significant in the univariable regression analyses were entered in multiple regression models.

**Fig. 1:**
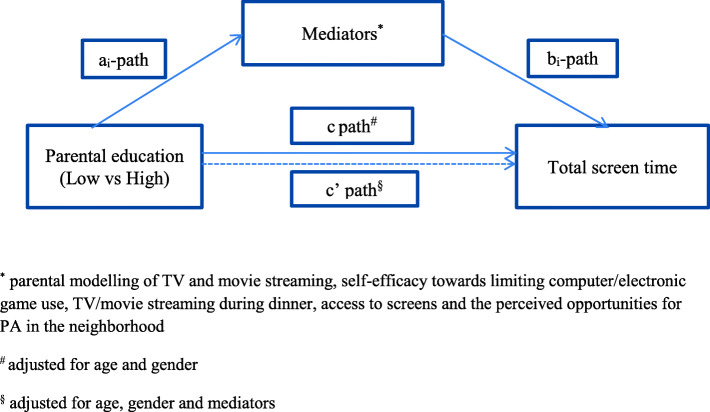
Mediation model

Mediation analyses were thereafter conducted. Figure 1 illustrates the multiple mediation model used. In the multiple mediation analysis, the ai-paths represent the association between parental education and each mediator. The bi-paths represent the association between the mediators and TST (adjusted for parental education and the other mediators). The c’ path represents the association between parental education and TST when adjusted for mediators. The c path represents the total effect of parental education on TST. Gender and age were controlled for in the analyses. Bootstrap corrected CIs were calculated for indirect effects (a*b). Bootstrapping (5000 samples) was conducted using the PROCESS macro 3.4. for SPSS by Andrew Hayes [22]. Analyses were conducted using SPSS version 25. The significance level was set to 0.05.

## Results

Table 1 shows the characteristics of participants. Fifty four percent of participants were females and 40% had parents with low education. Parental education was significantly inversely related to TST. There were socioeconomic differences in all three different screen based activities used to compute total screen time (results not shown). Mean scores for self-efficacy to limit computer/electronic game use, perceived neighborhood opportunities for PA and perceived neighborhood facilities were significantly higher for those with high parental education compared to those with low parental education. Mean scores for TV/movie streaming during lunch and dinner, parental modeling and access to screens were significantly lower for those with high parental education compared to those with low parental education.

**Table 1:** Characteristics of participants and parental educational differences

	Total sample (*n* = 702)^a^	Low parental education	High parental education	p**
Age (years)	13.64 (0.3)	13.66 (0.3)	13.63 (0.3)	0.33
Gender (% female)	54.0	54.9	53.3	0.70
Total screen time (hrs/wk)	36.34 (17.51)	41.14 (18.54)	33.24 (16.12)	< 0.001
Self-efficacy to limit TV/movie streaming	4.84 (3.28)	4.56 (3.46)	5.01 (3.14)	0.08
Self-efficacy to limit computer/electronic games	5.94 (3.91)	5.53 (4.12)	6.22 (3.74)	0.03
TV/movie streaming during breakfast	2.40 (1.35)	2.51 (1.38)	2.32 (1.32)	0.073
TV/movie streaming during lunch	2.34 (1.23)	2.45 (1.25)	2.25 (1.19)	0.030
TV/movie streaming during dinner	2.14 (1.26)	2.36 (1.30)	1.98 (1.18)	< 0.001
Co-viewing with parents	3.79 (3.31)	4.03 (3.75)	3.62 (2.94)	0.107
Parental modeling	3.27 (0.80)	3.42 (0.80)	3.19 (0.77)	< 0.001
Access to screens	1.93 (0.87)	2.07 (0.84)	1.84 (0.88)	0.001
Perceived opportunities for PA in neighborhood	4.04 (1.09)	3.91 (1.14)	4.10 (1.05)	0.028
Neighborhood facilities	3.65 (1.57)	3.41 (1.65)	3.75 (1.52)	0.006
Neighborhood safety	4.66 (0.79)	4.62 (0.78)	4.69 (0.81)	0.247

^a^*n* varies slightly between variables because of missing data

**p-value for differences between parental education groups (ANOVA and chi-squared test)

*PA* physical activity

Results of multiple linear regression analyses, adjusted for gender, age and parental education, showed that parental modelling of TV and movie streaming (B = 4.44 (CI: 2.90, 5.98)), TV/movie streaming during dinner (B = 1.10 (0.07, 2.13)) and access to screens (B = 1.81 (CI: 0.42, 3.20)) were positively related to TST. Self-efficacy towards limiting TV and movie streaming (B = − 0.62 (CI: − 1.03, − 0.21)), self-efficacy towards limiting computer/electronic game use (B = − 1.18 (CI: − 1.54, − 0.82)), and the perceived opportunities for physical activity (PA) in the neighborhood (B = − 1.39 (CI: − 2.47, − 0.31)) were inversely related to total screen time after adjusting for gender, age and parental education. The model explained 31% of the variability in TST (Table 2).

**Table 2:** Correlates of total screen time (hrs/week) among adolescents

	B and CI	*p* value
Self-efficacy to limit TV/movie streaming	- 0.62 (−1.03, − 0.21)	0.003
Self-efficacy to limit computer/electronic games	−1.18 (− 1.54, − 0.82)	< 0.001
TV/movie streaming during breakfast	0.76 (− 0.20, 1.71)	0.12
TV/movie streaming during lunch	1.01 (− 0.06, 2.08)	0.07
TV/movie streaming during dinner	1.10 (0.07, 2.13)	0.04
Co-viewing with parents	0.26 (−0.11, 0.64)	0.16
Parental modeling	4.44 (2.90, 5.98)	< 0.001
Access to screens	1.81 (0.42, 3.20)	0.01
Perceived opportunities for PA in neighborhood	−1.39 (−2.47, −0.31)	0.01

Results obtained from multiple linear regression analyses

Gender, age and parental education adjusted for in the analyses

*PA* physical activity

In the multiple mediation analyses, the factors found to mediate the association between parental education and TST were: parental modelling of TV and movie streaming (ab = − 1.10 (CI: − 1.76, − 0.34)), self-efficacy towards limiting the use of computer/electronic games (ab = − 0.81 (CI: − 1.69, − 1.39)), access to screens (ab = − 0.40 (CI: − 0.84, − 0.07), TV/movie streaming during dinner (ab = − 0.41 (CI: − 0.97, − 0.01) and perceived opportunities for PA in neighborhood (ab = − 0.29 (CI: − 0.78, − 0.01). Parental modeling and self-efficacy towards limiting the use of computer/electronic games were the strongest mediators (together mediated around 28% of the association between parental education and TST). Access to screens, TV/movie streaming during dinner and perceived opportunities for PA in neighborhood had weaker mediating roles (Table 3).

**Table 3:** Mediators of the association between parental education and total screen time among adolescents

	c-path	c’-path	a-path	b-path	ab
Parental modeling			−0.22(−0.34, − 0.09)	4.63 (3.10, 6.16)	−1.10 (− 1.76,-0.34)
Self-efficacy to limit electronic games			0.69 (0.09, 1.30)	−1.17 (− 1.53, − 0.80)	− 0.81(− 1.69, − 1.39)
TV/movie streaming during dinner			− 0.37 (− 0.57, − 0.17)	1.10 (0.08, 2.12)	−0.41(− 0.97, − 0.01)
Access to screens			− 0.21 (− 0.35, − 0.07)	1.93 (0.55, 3.32)	−0.40(− 0.84, − 0.07)
Perceived opportunities for PA in neighborhood			0.22 (0.04, 0.40)	−1.33 (− 2.41, − 0.24)	−0.29(− 0.78, − 0.01)
	−6.97 (−9.71, −4.22)	−3.39 (−5.89, − 0.89)			

Significant mediators shown

All paths were adjusted for gender and age

Ref. category: low parental education

*PA* physical activity

## Discussion

The study aimed to explore correlates of TST among adolescents and to assess their mediating role in the association between parental education and TST. Findings show that parental modelling of TV and movie streaming, TV/movie streaming during dinner and access to screens were positively related to TST. Self-efficacy towards limiting TV and movie streaming, self-efficacy towards limiting computer/electronic game use and the perceived opportunities for PA in the neighborhood were inversely associated with TST. All of these factors except self-efficacy towards limiting TV and movie streaming mediated the association between parental education and screen time.

Parental modeling [6–8, 23], access to electronic devices [7, 9] and eating meals in front of the TV [24] have previously been found to be associated with SB. The outcome of interest has however often been TV viewing and not TST, and more contemporary screen activities such as social media use were not included in most of these studies. Self-efficacy has been less explored in relation to its association with screen time, but is a widely documented predictor of health behaviors and is also emphasized in prominent theories of health behaviors such as the social cognitive theory [25]. The findings of the present study reflect its important role in influencing TST as well. The association between neighborhood PA opportunities and SB documented in this study contributes to the limited evidence in the literature. Perceived neighborhood safety was not associated with TST, in contrast to the findings of other studies that documented an impact of neighborhood safety on screen time. Timperio et al. found that neighborhood crime was associated with more time spent watching TV among adolescents [26]. Another study similarly documented that perception of low perceived neighborhood safety was associated with a higher screen time adolescent girls [27]. This could be due to the fact that parents might restrict PA outside the home when they perceive the neighborhood environment to be unsafe [28, 29]. However, the present study was conducted in a predominantly semi-rural area in Norway where safety concerns are low, potentially leading to little variability between participants. Indeed, the mean of the variable was high and showed a ceiling effect, which might make associations difficult to detect.

Several of the correlates of SB included in this study differed by SEP, reflecting individual, familial and perceived environmental exposures that vary by socioeconomic position. Socioeconomic differences in parental modeling, eating meals in front of the TV and access to screens (namely bedroom TV) among youth have previously been documented in a systematic review of the literature in which most studies used parental education as an indicator of SEP [13]. Self-efficacy to engage in healthier behaviors among adolescents such as healthier dietary behaviors has been found to differ by parental educational level [30, 31]; the same was found to be true for self-efficacy to limit screen activities. In addition, in the present study, perceived opportunities for PA in the neighborhood and neighborhood PA facilities were also found to differ by SEP, being more favorable among those with a higher SEP. There are multiple levels of influence on health behaviors, as postulated by the social ecological model [12]. The results of this study highlight differences at individual, interpersonal and perceived environmental levels that make it difficult for those with a lower socioeconomic position to engage in more favorable behaviors.

Most of the correlates that varied by socioeconomic position were found to mediate socioeconomic differences in TST. These factors represent important entry points for interventions to limit screen time and tackle socioeconomic differences. However, existing evidence from interventions and from qualitative studies indicate the multiple challenges that exist when trying to address screen time among youth. Systematic reviews of the literature indicate that the impact of interventions on reducing screen time among youth is either limited [32, 33] or absent [34]. Factors such as the strong habitual component of SB and the high accessibility and appeal of screen time (in particular in an age of high technological advances) have been incriminated for the lack of effect of interventions [32]. Systematic synthesis of qualitative evidence also indicates that screen time is an established norm among youth, which represents a significant obstacle for interventions [35]. Effective and multilevel approaches are thus needed to address screen time. Parental modelling was found to be an important correlate and an important mediator of socioeconomic differences in TST in the present study. Qualitative research evidence suggests that parents engage in screen-based SB even though they recognize that it is important to reduce the screen time of youth [35]. It is thus important to emphasize to parents that efforts aimed at reducing screen time also require their active participation. The findings of the study also suggest that restricting access to screens could be a potential strategy to reduce screen time and related social inequalities among adolescents. However, qualitative evidence indicates that restricting such access, in particular the removal of bedroom TV, can be met with substantial opposition namely among adolescents [36]. Participants also indicated that not putting a TV in the child’s bedroom would be easier than removing one that was already in place [36]. These findings indicate that avoiding excessive exposure to screens should start early in childhood. Screen viewing during meals, in particular during dinner, was also found to be associated with a higher screen time and mediated socioeconomic differences in screen time. The literature shows that parental education is positively related to having family meals [37], which is inversely related to TV viewing during meals [38], which might in part explain the findings of the study. One of the strategies to reduce screen use during meals can therefore be the promotion of family meals, in particular among those with a lower socioeconomic position.

The perceived opportunities for PA in the neighborhood were also found to predict screen time and mediate socioeconomic differences. SBs and PA are not opposite sides of the same coin. However small inverse associations between the behaviors are documented in the literature [39]. Indeed, if no social or physical opportunities for engaging in PA around the home and in the neighborhood exist, the alternative option in this day of developing social media and technological development is likely going to be more engagement in screen time. In this regard, research evidence shows that youth report higher involvement in screen activities when they perceive a lack of peer social support networks [35]. In line with the findings of the study, qualitative interviews with both parents and adolescents indicate that a lack of physical opportunities to engage in PA near the home can result in a higher screen time [35]. These results reflect that efforts by policy makers towards improvement of neighborhood PA opportunities would also benefit youth through the reduction of screen time, in addition to promoting PA. Such efforts might be particularly relevant for those with a lower socioeconomic position among whom alternative leisure-time activities that require resources are likely to be more limited.

### Strengths and weaknesses

Screen-based SBs tend to co-occur (multitasking), thus TST is likely to overestimate the time that adolescents spend on screen-based activities. However, interventions are likely to target TST in general and not single screen-based activities. Thus, TST was used as an outcome in the present study. However, future studies with measures accounting for the multitasking behavior of adolescents are warranted, as also highlighted in a recent systematic review of the literature [1]. The use of self-reported measures is associated with problems of validity and reliability. In general, the more unreliable the measures, the higher the chance of Type II errors; that is not finding differences and associations that in fact exist. Therefore, differences and associations in the study were probably underestimated rather than overestimated. Education was the only indicator of SEP used in the current study. It was reported by parents, resulting in a low rate of missing data. Parental reports of education are also likely to be more accurate than adolescent reports. However, different indicators of SEP can reflect different social, material and financial assets that can influence behavior. Thus, future studies including other indicators of SEP are warranted to explore whether the association between SEP and TST is indicator-specific. The age range of participants is narrow and the results might thus namely be applicable to younger adolescents, although the correlates and mediators identified are relevant for older adolescents as well.

The inclusion of a broad range of SBs including behaviors relevant for contemporary youth is a strength of the study. Several modifiable correlates and mediators were also included, addressing a gap in the existing literature. The sample size was relatively large, and a good response rate was achieved.

## Conclusions

The study identified several modifiable factors at the individual, interpersonal and perceived environmental levels that can be targeted in interventions aimed at decreasing screen time among youth in general and among those with a low socioeconomic position in particular. More correlates of TST and more mediators of socioeconomic differences in screen time should be explored in future studies. These studies should be complemented with qualitative in-depth exploration of the multiple interacting factors that act as barriers and facilitators of screen time, in particular among those with a lower socioeconomic position.

## Data Availability

The datasets used in the current study are available from the corresponding author on reasonable request.

## Abbreviations

SEP: Socioeconomic position
SB: Sedentary behavior
TST: Total screen time
TV: Television
